# Ada Lovelace Day 2021: Navigating a career in STEM. An interview with Jacqueline Gottlieb, Kirsty Bannister and Natasha Pushkin

**DOI:** 10.1038/s42003-021-02698-7

**Published:** 2021-10-12

## Abstract

October 12th is Ada Lovelace Day. Every year on this day we celebrate the achievements of women in science, technology, engineering and maths (STEM). Although we have made progress in terms of gender equality in STEM, hurdles are still faced and changes are still needed. We spoke to Professor Jacqueline Gottlieb, Dr Kirsty Bannister and Natahsa Pushkin about their journeys in STEM in what are still male-dominated disciplines.

Professor Jacqueline Gottlieb came to the US from Israel to pursue an undergraduate degree at MIT. She ended up majoring in Brain and Cognitive Science—which she states was the most exciting hot new topic back then—before going on to do a PhD at Yale. There, she became fascinated by the frontal cortex and did her dissertation on neural recordings of the frontal eye fields with Charlie Bruce. After a short foray on in vitro slice recordings in barrel cortex, she returned to in vivo recordings and completed her postdoctoral training at the NIH, eventually obtaining a faculty position at Columbia University. Throughout this time, her interests focused on the mechanisms of attention and decision making, leading to her current work on curiosity and information demand, which she sees as a big bridge between the two.

**Figure:**
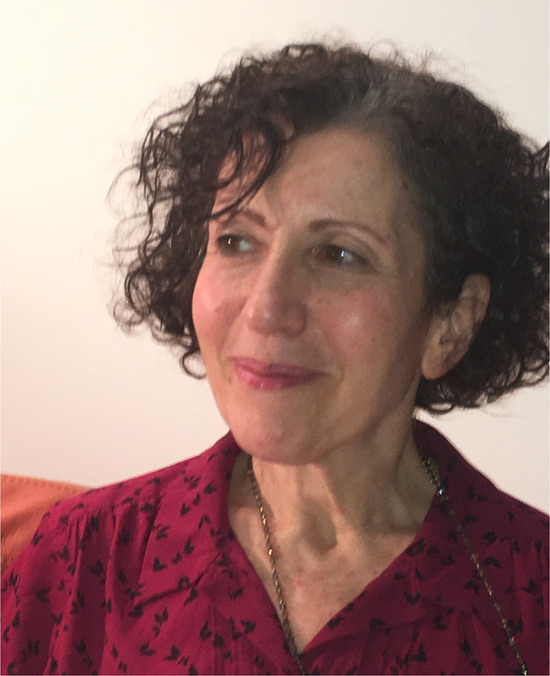
Jacqueline Gottlieb

Dr. Kirsty Bannister began her career in STEM in 2000 when she attended University College London to complete a BSc in Pharmacology. Having enjoyed the experience, she followed up with a Master of Research (Biomedical Integrative Sciences) and PhD (Epigenetic Mechanisms) from Imperial College London. Enthralled by the prospect of continuing to carry out scientific research, she returned to UCL in 2008 to begin a postdoctoral position in Professor Anthony Dickenson’s research group. She spent 9 years characterizing descending modulatory controls in health and disease before being appointed as a Lecturer at King’s College London in 2017. Now, as a Senior Lecturer, she runs a research group that bridges the gap between bench and bedside pain research. Specifically, her lab conducts exploratory experiments that seek to molecularly, anatomically and/or functionally define descending control pathways in healthy rodents and rodent models of chronic pain as well as in healthy human volunteers and chronic pain patients. Her funders include the Academy of Medical Sciences, the National Centre for the Replacement, Refinement and Reduction of Animals in Research, Parkinson’s UK and the MRC.

**Figure:**
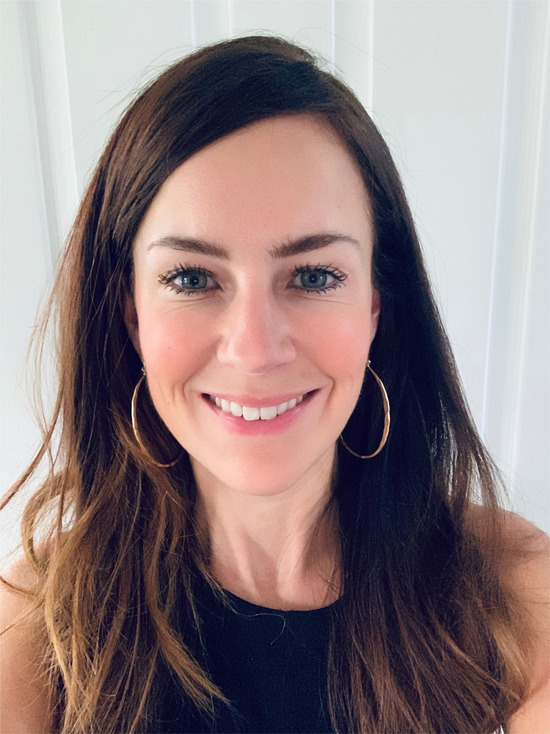
Kirsty Bannister

Natasha Pushkin graduated from the University of Kent, Canterbury with an MPhys in Astronomy, Space Science and Astrophysics. Her degree also included a year abroad in which she studied at Indiana University, Bloomington. She started with Airbus Defence and Space in 2009 as a Thermal Engineer on a two year graduate programme. She then progressed to Senior Thermal Engineer in 2013 and since 2016 has become a Propulsion Engineer. Day-to-day, Natasha uses a variety of internal and external tools to perform analyses that support the design of spacecraft propulsion systems. Her other tasks include supporting equipment procurement and testing. Through this, she works on projects that involve earth observation, such as how climate change affects our oceans, and exploratory missions such as Mars Sample Return.

**Figure:**
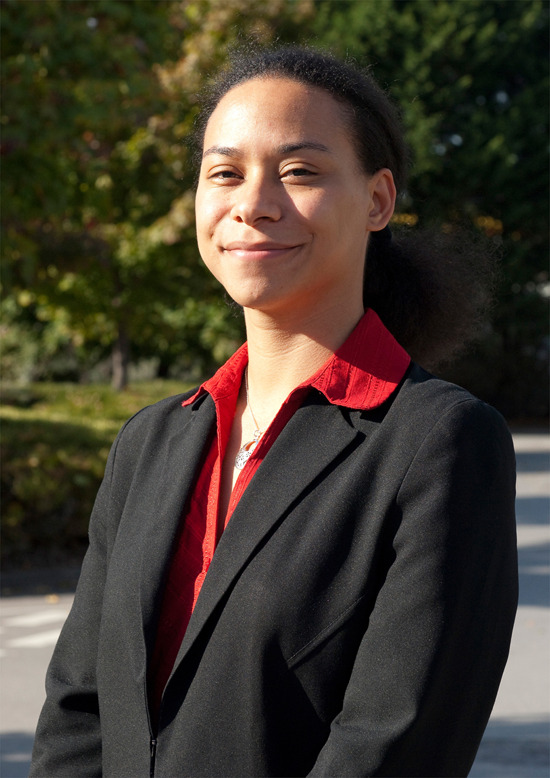
Natasha Pushkin

What first inspired you to pursue a career in STEM?

[JG]: I was very curious about how things work and particularly how systems learn and adapt. I vacillated at first between neuroscience and immunology, as I believe that the brain and the immune system are our two most intelligent systems. I also discovered that I loved working in labs—it was so exciting to understand how something works and then make it happen. The process of making a guess—a hypothesis—testing it out and obtaining an answer made me feel that I conversed directly with nature.

[KB]: I always loved science at school. My A level Biology teacher, a woman, was young, fun and wore clothes other than a white lab coat. Miss Hughes provided me with the first real confidence that women could be scientists too. Having also enjoyed Chemistry at A level, an undergraduate degree in Pharmacology seemed like an obvious choice. I started my first degree in 2000, and I haven’t looked back since. With each degree I gained a new respect and passion for basic science and now, running my own research group, I work hard to inspire other women that basic science is for them too.

[NP]: For me my career choices have always been about what I enjoy and what I was interested in. My job not only uses aspects of my education for interesting space topics but the projects also have practical applications.

What hurdles have you faced as a woman in STEM?

[JG]: At first, I was not aware that I was facing any hurdles. My mom and dad were physicians and I never thought there was something that I could not do if I wanted to. The realization that I needed to work much harder than others to receive recognition came gradually, as I advanced in my career. The biggest hurdle for me was simply being a minority. Not being part of the majority group—males—meant that I had fewer interactions, received fewer kudos and encouragement and was privy to fewer discussions and ideas. Sustained throughout the years, this translated to less recognition, fewer students, fewer high-impact papers and grants – in short, less of all the resources that help so much in research.

[KB]: It is undeniable that women are the minority when it comes to those who hold senior scientist positions in academia. In the early days of my career in STEM this influenced my long-term goals. For example, for many years it did not occur to me that I would ever become a Principal Investigator or a Professor. This psychological hurdle stopped me from applying for certain awards as I didn’t think that I fit the scientist mould. Subsequently, having children impacted my progression as a postdoctoral researcher (despite my incredibly supportive PI) as I had to miss conferences and key networking events. The hurdle of transitioning to a full-time working scientist with caring responsibilities at home was not easily overcome. The preconceptions that others hold regarding my understanding of the scientific subject matter that I am specialist in are not always favourable, and on more than one occasion at scientific meetings I have been assumed to be a waitress in the coffee break.

Who has been a female role model that has had the biggest impact on your career?

[JG]: Patricia Goldman Rakic was the chair of the Neurobiology Department at Yale when I was a PhD student. She had a passionate interest in the prefrontal cortex and was fiercely determined and focused on her research. At the same time, she was feminine and vulnerable. She tragically died in a car accident. I miss her. There would have been so many things I would have liked to talk to her about as I approached the career stage she was in.

[KB]: I am lucky enough to have had several. Professors Irene Tracey, Bridget Lumb and Annette Dolphin have each, at different timepoints in my career in STEM, offered me guidance, advice, and support. Their mentorship combined has given me the confidence to believe that I could work towards Professorship and that, despite being a woman, it is possible to succeed in this arena. Additionally, and perhaps most importantly, these women taught me that to succeed in science, you can and should cheerlead other women. Why be an obstacle when you can empower and uplift in a manner that causes no detriment to your own career?

[NP]: For me, there is nobody specific but I have a great amount of respect and admiration for the women at my company in the generation above. My generation has seen great improvements with respect to the representation of women in the field for which the road was paved very much by this generation before.

Professor Gottlieb, as a very senior scientist, how do you think things have changed for women in STEM over the years?

[JG]: Things changed a lot and for the better. As an undergraduate at MIT, it was common to find myself as the only woman in a lecture or classroom. As a PhD student, I was aware of only a handful (perhaps 3 or 4) prominent women neuroscientists. None of these women had children, making it seem that they had to choose between having a family or having a career. This is no longer the case, which is a tremendous advance.

What do you think still needs to change for women in STEM?

[KB]: Women are still under-represented at key national and international scientific congresses, and this is where I believe the biggest change is needed. If the young female scientists attending these meetings don’t see a representation of themselves on the podium, they miss out on the opportunity to imagine themselves in that position.

[JG]: Despite the increase in the number of women, the sociological barriers remain very real. Women are still being pushed down at every step and, as has been documented in many studies, this is often through a web of subtle and unconscious biases. I think there are two antidotes to this. First, continued increase in the representation of women will naturally bring women’s interests to the fore. Second, people need to be aware that everyone is biased toward the majority. It is a universal human tendency to ignore many things—the brain does this automatically and people who claim to be unbiased are actually the most biased. Acknowledging one’s limitations does not mean that one should feel bad or guilty about them (women and other minorities are biased too). It only means that we can remember to make some deliberate efforts to overcome unconscious biases. It is very important that these efforts do not sacrifice quality; one does not want to feel that they were favored just for being a woman. But just having humility and acknowledging that everyone can be biased can go a very long way. And it helps so much if this comes from the majority group (men). Having a man stand up for a colleague who was ignored in a faculty meeting (“that’s what she said!”) or for one who was passed over for a prize nomination (“I would like to talk about her findings a little more”) is such a powerful way to change perceptions and such an amazing way give women a confidence boost.

[NP]: Ultimately it comes down to trying to address why aren’t there more women in specific disciplines and then tackling the resulting answer. So I think in general, regardless of the field, a key change is having that discussion with young people and encouraging them to pursue their desired paths.

Interview conducted by Associate Editor Karli Montague–Cardoso for Ada Lovelace Day 2021

